# The fate of *Meconopsis* species in the Tibeto‐Himalayan region under future climate change

**DOI:** 10.1002/ece3.7096

**Published:** 2020-12-28

**Authors:** Wen‐Ting Wang, Wen‐Yong Guo, Scott Jarvie, Jens‐Christian Svenning

**Affiliations:** ^1^ School of Mathematics and Computer Science Northwest Minzu University Lanzhou China; ^2^ Key Laboratory of China's Ethnic Languages and Information Technology of Ministry of Education Northwest Minzu University Lanzhou China; ^3^ Department of Biology Center for Biodiversity Dynamics in a Changing World (BIOCHANGE) Aarhus University Aarhus C Denmark; ^4^ Department of Biology Section for Ecoinformatics & Biodiversity Aarhus University Aarhus C Denmark

**Keywords:** alpine plants, climate change, climate refugia, dispersal routes, species’ vulnerability

## Abstract

High‐mountain areas such as the Tibeto‐Himalayan region (THR) host cold‐adapted biota expected to be sensitive to anthropogenic climate change. *Meconopsis* is a representative endangered genus confined to alpine meadow or subnival habitats in the THR. We used climate‐niche factor analysis to study the vulnerability of ten *Meconopsis* species to climate change, comparing current climate (representative of 1960–1990) to future climate scenarios (2070: average 2061–2080). For these ten *Meconopsis* species, we then identified potential future climate refugia and determined optimal routes for each species to disperse to the proposed refugia. Our results indicate that for the ten *Meconopsis* species, the regions with low vulnerability to climate change in the THR are the central Qinghai‐Tibet Plateau, the Hengduan Mountains (HDM), the eastern Himalayas, and the West Qinling Mountain (WQL), and can be considered potential future climate refugia. Under future climate change, we found for the ten *Meconopsis* species potential dispersal routes to three of the four identified refugia: the HDM, the eastern Himalayas, and the WQL. Our results suggest that past refugia on the THR will also be the future climate refugia for the ten *Meconopsis* species, and these species may potentially persist in multiple future climate refugia, likely reducing risks from climate change. Furthermore, climate change may affect the threat ranking of Red Listed Species for *Meconopsis* species, as Least Concern species were estimated to become more vulnerable to climate change than the only Near Threatened species.

## Graphical abstract

Under future climate change, *Meconopsis* species may persist in multiple refugia on the Tibeto‐Himalayan region, with these areas also coinciding with past glacial refugia. Climate change may affect the threat ranking of Red Listed Species, as Least Concern species were estimated to be more vulnerable to climate change than Near Threatened species.

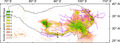

## 1 INTRODUCTION

Anthropogenic climate change has already had profound effects on global biodiversity, and will likely have even stronger impacts in the future (Bellard et al., 2012; Dawson et al., 2011; Thuiller et al., 2005). Alpine ecosystems are particularly sensitive to climate change because their biota is generally limited by low temperatures (Vanneste et al., 2017). Notably, ongoing climate change is driving an accelerated increase in species richness on mountain summits (Steinbauer et al., 2018). Furthermore, ongoing climate change is strongly reshaping the distribution of montane plants even at low latitudes (Grytnes et al., 2014), for example, vegetation on the Chimborazo volcano in Ecuador has undergone a strong upslope shift in the past two centuries (Morueta‐Holme et al., 2015). Another mountain system affected by climate change is the Tibeto‐Himalayan region (THR), which includes the world's largest and highest plateau, the Qinghai‐Tibet Plateau (QTP proper), and two important alpine biodiversity hotspots, the Hengduan Mountains (HDM) and the Himalayas (Huang et al., 2008; Muellner‐Riehl, 2019). The THR is regarded as a region sensitive to climate change (Duan & Xiao, 2015; Yao et al., 2000), with surface air temperature predicted to increase at 1.5 times the average global warming rate (Zhang et al., 2013). The species currently found in the THR will likely face huge challenges from future climate change, with their fate being of widespread concern (Liao et al., 2020; You et al., 2018).

Refugia with complex topography may buffer against the effects of climate change (Bátori et al., 2016; Scherrer & Körner, 2011) and allow for the local persistence of species through successive periods of climate change (Ronikier et al., 2012). For example, the Karkonosze Mountains in Poland have been shown to be an important refugium for Central European mountain flora (Suchan et al., 2019). Past refugia often have geographical features that allowed species to persist through extreme climate events (Avise, 2008; Hampe & Jump, 2011; Hannah et al., 2002). Phylogeographical studies suggest that extreme climate fluctuations during the Quaternary, for example, between the Last Interglacial (LIG) and the Last Glacial Maximum (LGM), have had a weaker filtering effect (extinction) in the THR than some other mountain systems, for example, the European mountains and North American mountains (Li et al., 2010; Muellner‐Riehl et al., 2019; Peng et al., 2007; Wang et al., 2005). The weaker filtering effect in the THR is likely due to a buffering effect from an exceptionally large elevational range (Li et al., 2010; Wang et al., 2005). In the Quaternary, long‐term refugia existed in the THR, allowing species to persist through at least one glacial‐interglacial cycle (Liu, Duan et al., 2014; Peng et al., 2007; Sun et al., 2010; Tang & Shen, 1996; Wang et al., 2009). Recently, some studies have suggested that long‐term refugia are likely to keep on acting as such under future climate change (Ashcroft, 2010; Ashcroft et al., 2012; Gavin et al., 2014). Hence, these long‐term refugia, including the HDM region (Chen et al., 2008; Zhang et al., 2005), may represent the best chance for species currently found in the THR to survive in the future.

Responses of plant population to past climate change can be attributed to dispersal, in situ adaptation, and extinction (Aitken et al., 2008; Christmas et al., 2016). The Quaternary record suggests that dispersal has been a common response of species to past climate change to avoid extinction (Huntliey, 1995). At a global scale, species are generally expected under future climate change to disperse poleward and upward (Parmesan & Yohe, 2003), although a recent study suggested some species may respond with omnidirectional range shifts at smaller spatial scales (Lenoir & Svenning, 2015). Moreover, although dispersal capacity is key for species to track future climates, many alpine plants have a low capacity for long‐distance range shifts due to their limited seed dispersal ability (Morgan & Venn, 2017). The availability of suitable habitat along dispersal routes is important for species to successfully shift to habitat that will remain climatically suitable in the future (Skov & Svenning, 2004). Habitat fragmentation, farming practices, and urbanization will reduce the capacity of many species to cope with climate change (Putten et al., 2010). For example, Kuhn et al. (2016) suggested that the distribution ranges of 25 submountainous plant species currently occurring throughout Europe will fragment to mountain ranges in southern and northern Europe due to future climate change. In the THR, the high geodiversity is characterized by a large mountain system and plateau surfaces that form a complex climatic environment. A previous study by Wang et al. (2009) suggested four refugia on the THR during the LGM for the alpine plant species *Aconitum gymnandrum*. Similarly, we expect there to be more than one climate refugium on the THR under near‐future climate change, and that species with large current distributions may persist in multiple refugia in the future.

The vulnerability of species to climate change, defined as the tendency to be adversely affected by climate change, can provide valuable information to understand climate‐related risks posed to species (Füssel & Klein, 2006; Stanton et al., 2015). An important challenge for conservation in the THR is therefore to identify species vulnerable to climate change. Recently, Rinnan and Lawler (2019) developed the climate‐niche factor analysis (CNFA) method to assess the vulnerability of species to climate change. This new method expands on the earlier ecological‐niche factor analysis (Basille et al., 2008; Hirzel et al., 2002), and can provide spatially explicit insights into geographic patterns of climate change vulnerability with presence‐only data (Rinnan & Lawler, 2019). The CNFA method is also able to make direct comparisons of climate change vulnerability among species. Compared with previous correlative approaches used to assess the vulnerability of species to climate change (Cole et al., 2011; Vieilledent et al., 2013), CNFA avoids uncertainties arising from differences in methods and models used to predict species distribution (Pacifici et al., 2015; Pearson et al., 2006; Rinnan & Lawler, 2019). Moreover, unlike trait‐based vulnerability assessment approaches (Foden et al., 2013; Williams et al., 2008), CNFA is not limited by gaps in knowledge of individual species’ traits (Foden et al., 2013; Rinnan & Lawler, 2019; Thomas et al., 2011). In this study, we assess the vulnerability of species in the *Meconopsis* genus to future climate change. The *Meconopsis* genus belongs to Papaveraceae family, with ~60 species confined to alpine meadow or subnival habitats in the THR, and is a symbol of the Himalayan alpine flowers (Liu, Liu et al., 2014; Yang et al., 2012). A recent study by He et al. (2019) modeled the potential distribution of *Meconopsis* species under climate change through ecological niche models. Building on this, we used CNFA to assess the vulnerability of *Meconopsis* species to climate change, identifying potential future climate refugia. We also investigate the impact that habitat fragmentation might have on the ability of *Meconopsis* species to disperse to the identified refugia in response to climate change.

## 2 MATERIALS AND METHODS

### 2.1 Occurrence data and species ranges

**FIGURE 1:**
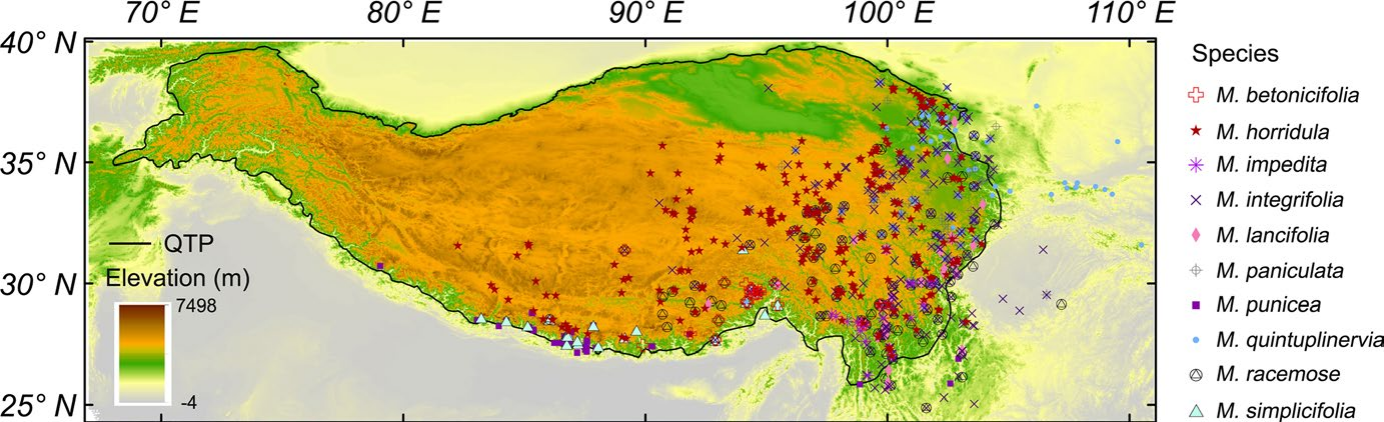
Occurrence data for ten *Meconopsis* species studied in the Tibeto‐Himalayan region (THR) and adjacent areas (*N* = 856 for the ten species). The number of records per species can be found in Table S1

To obtain occurrence data for *Meconopsis* species found in the THR and adjacent regions, we used records from the Chinese Virtual Herbarium (CVH: http://www.cvh.org.cn/), the Global Biodiversity Information Facility (GBIF: http://www.gbif.org/), and the published literature (He et al., 2019). We only used occurrence data collected after 1950 and removed duplicate records found within a 5‐km diameter. For subsequent analyses, we retained occurrence records for ten species of *Meconopsis*, with the numbers of records per species ranging from 17 to 228 (see Figure 1 for the distributions and Table S1 for the number of records per species). According to the Red List of Chinese plants database (http://www.chinaplantredlist.org/), the two widely distributed species of the *Meconopsis* genus found on the THR, *M. horridula* and *M. integrifolia*, are evaluated as Near Threatened (NT) and Least Concern (LC), respectively. The four quite narrowly distributed species found in southeast QTP, *M. betonicifolia*, *M. impedita*, *M. paniculata,* and *M. simplicifolia*, are evaluated as LC. The remaining four species, *M. racemose*, *M. quintuplinervia*, *M. punicea, and M. lancifolia,* are also evaluated as LC.

We estimated ranges for the ten *Meconopsis* species by constructing alpha hulls with the retained occurrence records based on the computational geometry method of Edelsbrunner et al. (1983), as implemented with the “ashape” function of the alphahull package (Pateiro‐López & Rodríguez‐Casal, 2010) in the programming language R (ver. 3.5.1; R Core Team, 2018). Estimates of species range size by the alpha hull method are rarely affected by sampling bias, and overestimation of range size is reduced by adjusting the alpha level (Burgman & Fox, 2003). Following the International Union for Conservation of Nature Standards and Petitions Working Group (2008), we applied an alpha level of 2 degrees when estimating each species alpha hull range. We also made a 10‐km buffer around disjunct records not included in the alpha hull range estimation, and included them in each species estimated range. To exclude unsuitable areas from estimated ranges, we used current land cover maps to remove areas covered by cities, water bodies, and permanent ice and snow cover. The current land cover maps had a spatial resolution of ~300 m and were from GlobCover products (ESA 2010 and UCLouvain, http://due.esrin.esa.int/page_globcover.php).

### 2.2 Bioclimatic variables

Bioclimatic variables of current climate (representative of 1960–1990) and future climate (2070: average of 2061–2080) were downloaded from the WorldClim database v1.4 (http://www.worldclim.org/) at a 2.5 arc‐min spatial resolution (Hijmans et al., 2005). To assess collinearity of the 19 current bioclimatic variables from WorldClim, we used a pairwise Pearson's correlation test (Table S2). We retained for subsequent analyses nine bioclimatic variables with Pearson's correlations <0.8 (Table 1). For future climates, we used an ensemble method of six global climate models (GCMs) due to climate uncertainty (Baker et al., 2016). The six GCMs used were ACCESS1‐0, HadGEM2‐AO, CCSM4, IPSL‐CM5A‐LR, MIROC5, and MRI‐CGCM3. For the future climate ensembles, we used two representative concentration pathways (RCPs) for prescribed greenhouse gas emissions: (1) RCP 4.5, which represents a medium CO_2_ emissions with the peak of global annual greenhouse gas emissions around 2040, followed by decline; and (2) RCP 8.5, which represents high CO_2_ emissions, with CO_2_ emissions continuing to rise throughout the 21st century (Harris et al., 2014; Meinshausen et al., 2011; van Vuuren et al., 2011).

**TABLE 1:** Bioclimatic variables used in climate change vulnerability assessments of ten *Meconopsis* species found in the Tibeto‐Himalayan region

Variable	Meaning of variables
Bio02	Mean Diurnal Range (Mean of monthly (Maximum Temperature of Warmest Month ‐ Minimum Temperature of Coldest Month))
Bio03	Isothermality (Bio02/Bio07 × 100)
Bio07	Temperature Annual Range (Maximum Temperature of Warmest ‐ Month Minimum Temperature of Coldest Month)
Bio08	Mean Temperature of Wettest Quarter
Bio09	Mean Temperature of Driest Quarter
Bio15	Precipitation Seasonality (Coefficient of Variation)
Bio16	Precipitation of Wettest Quarter
Bio17	Precipitation of Driest Quarter
Bio19	Precipitation of Coldest Quarter

### 2.3 Climate‐niche factor analysis

Species’ vulnerability can be thought of as a function of both extrinsic (exposure) and intrinsic (sensitivity and adaptability) traits (Pacifici et al., 2015). Exposure is the magnitude of climate change within the species’ geographic range (Williams et al., 2008). Sensitivity is the persistence ability of a species, determined by the climatic conditions of its habitat, while adaptability is the inherent ability of a species to tolerate climate change (Turner et al., 2003; Williams et al., 2008).

#### 2.3.1 Quantifying species niches

To quantify each species niche, we compared the species distribution in ecological space with the global distribution of available environmental conditions (Hirzel et al., 2002; Rinnan & Lawler, 2019). Following Rinnan and Lawler (2019), we quantified two aspects of a species’ niche: (1) the marginality, which is the niche centroid distance between the species distribution and the global distribution, and (2) the specialization, which is the ratio of size of the species niche to that of the global distribution. To define the global distribution in our study, we used the combined range of the ten *Meconopsis* species in the THR and adjacent regions as *N* cells, that is, the extent of the combined range was from 67°E to 111°E and from 24°N to 40°N. For the distribution of each *Meconopsis* species with *Ns* cells, we used both estimated ranges and occurrence records to represent each species distributions, and ran CNFA for both to verify the robustness of our results. For multi‐dimensional ecological space composed of bioclimatic variables with *P* dimensions, the components of marginality or specialization are defined as the marginality factors (m*_j_*) and the specialization factors (u*_j_*_1_, u*_j_*_2_,…,u*_jP_*_‐1_), respectively.

#### 2.3.2 Sensitivity, exposure, and vulnerability to climate change

We obtained the sensitivity factor through the marginality factor and specialization factor for each bioclimatic variable. We first normalized the vector (m*_j_*, u*_j_*_1_, u*_j_*_2_,…,u*_jP_*_‐1_) to ($w_{j1}$, $w_{j2}$,…,$w_{\mathrm{jP}}$). We then calculated the sensitivity factor *s_j_* corresponding to each bioclimatic variable *j* as $\sum_{k=1}^{P}w_{\mathrm{jk}}\rho_{k}$, where $\rho_{1}$ is the amount of specialization on the marginality axis, and $\rho_{k}\left(k>1\right)$ is the amount of specialization expressed on the specialization axis. For the global distribution, the predicted sensitivity of cell *i* is $\sigma_{\mathrm{Gi}}=1/P\sum_{j=1}^{P}\left(z_{\mathrm{ij}}‐m_{j}\right)s_{j}$, where $z_{\mathrm{ij}}$ represents the value of current bioclimatic variable *j* at location *i*. The overall sensitivity, $S=\sqrt{1/P\sum_{j=1}^{P}s_{j}}$, can then be used to compare the sensitivity between different species. The higher the overall sensitivity of a species, the more vulnerable it is to climate change (Rinnan & Lawler, 2019).

To calculate a species exposure to climate change, we used a dissimilarity measure of current and future climate within the species range. The departure factor is $d_{j}=\sum_{i=1}^{N}p_{j}\left|g_{\mathrm{ij}}‐z_{\mathrm{ij}}\right|$, where $g_{\mathrm{ij}}$ represents the value of future bioclimatic variable *j* at location *i*, and $p_{i}$ is the habitat utilization at location *i*. For the global distribution, the predicted exposure of cell *i* is $\delta_{\mathrm{Gi}}=\sum_{j=1}^{P}\left|g_{\mathrm{ij}}‐z_{\mathrm{ij}}\right|d_{j}$. Then the overall exposure is $D=\sqrt{\sum_{j=1}^{P}d_{j}^{2}}$. The higher the overall exposure of a species, the greater the departure of its habitat from current climate to future climate.

To calculate each species vulnerability to climate change, we combined sensitivity and exposure. To do this, we calculated the vulnerability factor $v_{j}$ for each bioclimatic variable *j* as $\sqrt{\left(1+d_{j}\right)s_{j}}$, and the predicted vulnerability of cell *i* for the global distribution as $\nu_{\text{Gi}}=\sqrt{\sigma_{\text{Gi}}\delta_{\text{Gi}}}$. Thus, the overall vulnerability is $V=\sqrt{1/P\sum_{j=1}^{P}v_{j}}$. Adaptability was not considered as climatic niche evolution is slower than the rate of climate change (Quintero & Wiens, 2013).

When the above steps are followed to calculate species’ sensitivity, exposure, and vulnerability, they constitute a climate‐niche factor analysis (CNFA). We implemented the CNFA method with the “cnfa” function of package CENFA (Rinnan, 2018) in R. We also used the “predict” function in the CENFA package to evaluate the spatial sensitivity, exposure, and vulnerability of each of the ten *Meconopsis* species to climate change. Maps of spatial sensitivity, exposure, and vulnerability were generated with ArcGIS 10.2 (ESRI, 2014).

### 2.4 Climate refugia and dispersal routes

In ecological and conservation planning, vulnerability is used to quantify the impact of threats on species extinction. Areas with low vulnerability under climate change are considered to be suitable in the future. We used thresholds to binarize the vulnerability estimates for each of the ten *Meconopsis* species from the CNFA analyses, with 0 indicating unsuitable areas and 1 indicating suitable areas. We then summed the suitable areas for each species to obtain values between 0 and 10. The higher the value, the greater number of species expected to survive in an area under climate change. We regarded areas with higher values as future climate refugia. Because no guidelines currently exist to determine thresholds for refugia from CNFA analyses, we tested the spatial vulnerability of each species using three quantiles of 1/20, 1/10, and 1/5. We also assessed if refugia obtained using these three quantiles were in the same approximate position.

For a species to arrive at climate refugia under future change, it depends on both their dispersal ability and the availability of suitable habitat along dispersal routes (Skov & Svenning, 2004). To establish a proxy of habitat availability, we used current land cover classes and occurrence records for the ten *Meconopsis* species. We then quantified the ratio of occurrence records for each land cover class to the total number of occurrence records as habitat availability, and simulated dispersal rates dropped sharply when habitat availability was below 25% (Collingham & Huntley, 2000). The land cover classes we used those defined by the United Nations Land Cover Classification System, with the ratio of the occurrence records on each land cover class to the total number of occurrence records shown in Table S3. As the remoteness of the THR will likely limit human‐induced land use and land cover modification in the future, we assumed the state of land cover will also be relatively stable. We therefore used current land cover to calculate habitat availability and then simulated dispersal resistance. We assumed dispersal resistance would approach zero when habitat availability was close to 100% (Skov & Svenning, 2004) and would increase exponentially to infinity when habitat availability approaches zero (Collingham & Huntley, 2000).

To determine optimal dispersal routes from current distributions to potential future climate refugia, we conducted least‐cost path analyses using Cost Weighted Distance mapping and the Shortest Path function as implemented in ArcGIS 10.2 (ESRI, 2014). For each species, we used occurrence records (Figure 1) as possible starting points and potential future climate refugia as destinations. We calculated dispersal resistance as the path cost and obtained the least‐cost paths as potential dispersal routes for each of the ten *Meconopsis* species.

## 3 RESULTS

### 3.1 Sensitivity, exposure, and vulnerability of ten *Meconopsis* species

Overall vulnerabilities from the CNFA analyses for both occurrence records and estimated alpha hull ranges show a significant linear relationship at the species level (*p*‐value < .05, *R^2^* = .52; Figure S1). The Pearson's correlation for spatial vulnerability for each species based on occurrence records and alpha hull ranges was >0.9 (Table S4). Therefore, we subsequently only report CNFA results based on the alpha hull ranges.

The CNFA results show most *Meconopsis* species are mainly sensitive to precipitation of driest and coldest quarters (Bio17 and Bio19) (Figure S2a). Moreover, their ranges consistently exhibit high departure (future deviations from current levels) of mean temperature during the driest and wettest quarters, but low departure of precipitation during the driest and coldest quarters (Figure S2b,c). Therefore, *Meconopsis* species generally exhibit equally high vulnerability to the mean temperature of wettest and driest quarters and precipitation of driest and coldest quarters under climate change (Figure S2d,e). The departure (Figure S2b,c) and vulnerability (Figure S2d,e) in bioclimatic variables show similar patterns for the two CO_2_ emission scenarios of RCP 4.5 and RCP 8.5.

**FIGURE 2:**
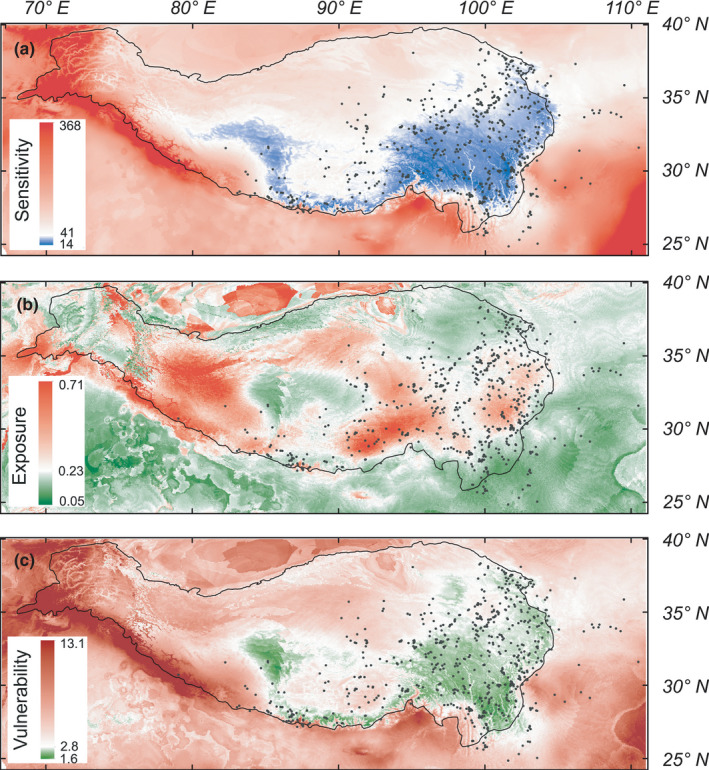
Mean of predicted sensitivity (a), exposure (b), and vulnerability (c) of ten *Meconopsis* species found in the Tibeto‐Himalayan region and adjacent areas under future climate for the year 2070. The future climate scenario was estimated from an ensemble of six global climate models projections under the representative concentration pathway (RCP) 4.5. The black line indicates the Tibeto‐Himalayan region. The black dots represent occurrence records for the ten *Meconopsis* species. For RCP 8.5, see Figure S3

**FIGURE 3:**
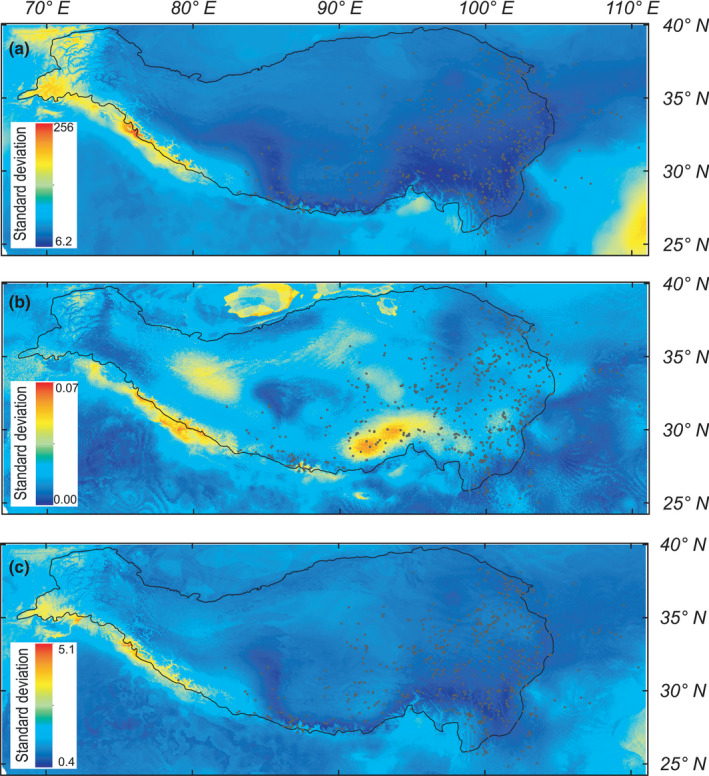
Standard deviation of predicted sensitivity (a), exposure (b) and vulnerability (c) of ten *Meconopsis* species found in the Tibeto‐Himalayan region and adjacent areas under future climate for the year 2070. The future climate scenario was estimated from an ensemble of six global climate models projections under the representative concentration pathway (RCP) 4.5. The black line indicates the Tibeto‐Himalayan region. The black dots represent occurrence records of the ten *Meconopsis* species. For RCP 8.5, see Figure S3

**FIGURE 4:**
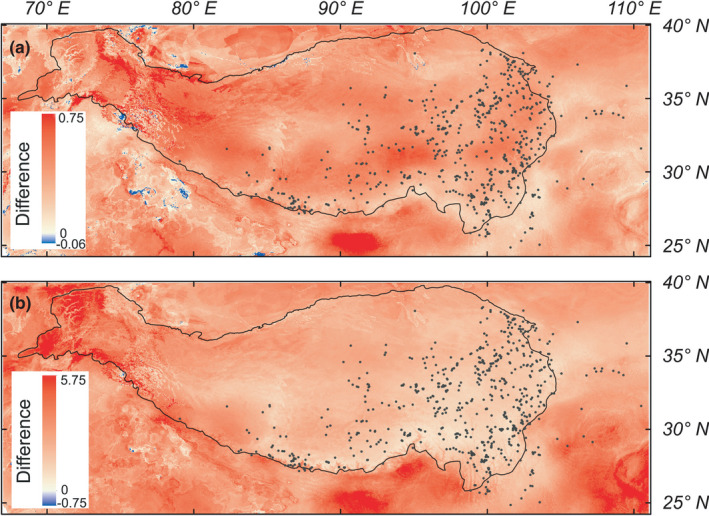
Difference in the mean of predicted exposure (a) and vulnerability (b) of ten *Meconopsis* species found in the Tibeto‐Himalayan region and adjacent areas under future climate for the year 2070. The future climate scenarios were estimated from an ensemble of six global climate models projections under the representative concentration pathways (RCPs) 8.5 and RCP 4.5. The black line indicates the Tibeto‐Himalayan region. The black dots represent occurrence records of the ten *Meconopsis* species

Because most of the ten *Meconopsis* species exhibit similar sensitivity, exposure, and vulnerability to the bioclimatic variables (Figure S2), we also calculated the mean and standard deviation for spatial sensitivity, exposure, and vulnerability, respectively. For the ten *Meconopsis* species, spatial sensitivity, exposure, and vulnerability had a low standard deviation (Figure 3, Figure S3c,d). Hence, mean spatial sensitivity, exposure, and vulnerability represent common characteristics. In order to show the impact of future climate change on the habitat of the ten *Meconopsis* species, we next calculated the mean sensitivity, exposure, and vulnerability values of occurrence records for the ten species from the global spatial sensitivity, exposure, and vulnerability, and used that to display interval values (Figure 2, Figure S3a,b). Figure 2a shows that the west‐central QTP proper, eastern Himalayas, and southeast THR are less sensitive to climate change for the ten *Meconopsis* species than other regions. Under RCP 4.5, most areas of the THR will experience high exposure to climate change, with these areas encircling the majority of the THR (Figure 2b). The ten *Meconopsis* species will experience high climatic exposure in the western and eastern regions of the HDM, while the regions with low exposure and low sensitivity to climate change are the central QTP proper, the eastern Himalayas and the central HDM (Figure 2a,b). The spatial vulnerability for the ten *Meconopsis* species is high in the west of the THR and low in the southeast of the THR. For most of the *Meconopsis* species, low‐vulnerability regions mostly surround the current area of the THR. Regions with low vulnerability are the central QTP proper, the HDM, the eastern Himalayas, and the West Qinling Mountain (WQL) (Figure 2c). The spatial exposure and vulnerability for the ten *Meconopsis* species under RCP 8.5 show a similar pattern for RCP 4.5 (Figure S3a,b). However, the high CO_2_ emission increases the climatic exposure in the THR (Figure 4a), leading to an increase in vulnerability for the ten *Meconopsis* species (Figure 4b).

Among the ten *Meconopsis* species, *M. lancifolia* has the highest overall sensitivity. In contrast, the overall sensitivity for *M. paniculata*, *M. simplicifolia*, *M. integrifolia*, and *M. horridula* is less than half of *M. lancifolia* (Table 2). For the ten *Meconopsis* species, the exposure under RCP 8.5 is higher than for RCP 4.5; hence, the vulnerability under RCP 8.5 is also higher than those under RCP 4.5 (Table 2). Compared with the other *Meconopsis* species, *M. lancifolia* has the highest overall vulnerability to climate change, followed by *M. betonicifolia*, while *M. paniculata* shows the least overall vulnerability. The vulnerability for six of the nine LC *Meconopsis* species is higher than that for the only NT species *M. horridula* (Tables 2, Table S1). The different CO_2_ emission scenarios do not change the vulnerability ranking for the ten *Meconopsis* species (Table 2).

**TABLE 2:** Overall sensitivity (Sens.), exposure (Exp.), and vulnerability (Vul.) of ten *Meconopsis* species in the Tibeto‐Himalayan region and adjacent areas under future climate for the year 2070. The future climate scenarios were estimated from ensembles of six global climate models projections under the representative concentration pathways (RCPs) 4.5 and 8.5

Species	Sens.	Exp. (RCP 4.5)	Exp. (RCP 8.5)	Vul. (RCP 4.5)	Vul. (RCP 8.5)
*M. paniculata*	6.38	0.52	0.71	2.46	2.49
*M. simplicifolia*	6.45	0.49	0.64	2.57	2.60
*M. integrifolia*	7.42	0.47	0.65	2.70	2.73
*M. horridula*	7.18	0.51	0.71	2.71	2.74
*M. racemose*	8.29	0.49	0.67	2.86	2.89
*M. quintuplinervia*	10.96	0.49	0.69	3.31	3.35
*M. punicea*	11.86	0.50	0.70	3.44	3.48
*M. betonicifolia*	12.52	0.63	0.79	3.56	3.59
*M. impedita*	14.31	0.47	0.63	3.69	3.73
*M. lancifolia*	14.86	0.45	0.62	3.81	3.85

### 3.2 Refugia and potential dispersal routes of ten *Meconopsis* species

**FIGURE 5:**
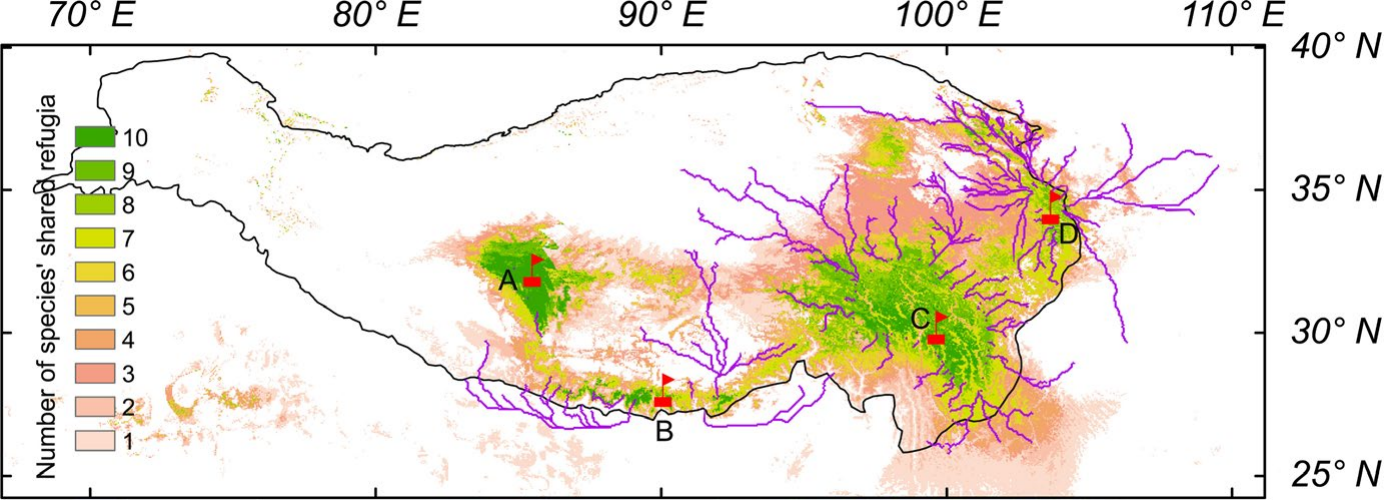
Potential future climate refugia and dispersal routes of ten *Meconopsis* species in the Tibeto‐Himalayan region and adjacent areas under future climates for the year 2070. The future climate scenario was estimated from an ensemble of six global climate models projections under the representative concentration pathway (RCP) 4.5. The climate refugia shown were obtained with a spatial vulnerability threshold equal to the 1/10 quantile. Red flags (a–d) represent approximate locations of the potential climate refugia, a: the central Qinghai‐Tibet Plateau, b: the eastern Himalayas, c: the Hengduan Mountains, d: the West Qinling Mountains. The solid purple lines show potential dispersal routes (from least‐cost path analyses, given current land use, and land cover constraints) for *Meconopsis* species to these potential climate refugia. The black line indicates the Tibeto‐Himalayan region

The three quantiles of spatial vulnerability used for each *Meconopsis* species identified climate refugia in the central QTP proper, the eastern Himalayas, the WQL, and the HDM. While the thresholds altered the total area of the identified refugia, the central locations were found to be similar for the three thresholds, with the results being consistent for both CO_2_ emission scenarios (Figure 5, Figure S4).

For dispersal of *Meconopsis* species from current locations to potential climate refugia under future climate change as determined by the least‐cost paths analyses, species currently distributed in the northeast of the THR would disperse to WQL, species in the southeastern margins of THR would disperse to the HDM, and species found between the central QTP proper and the HDM would disperse to either the HDM or the eastern Himalayas (Figure 5). The dispersal routes for species currently near the Himalayas all lead to refugium in the eastern Himalayas (Figure 5). Importantly, although the central QTP proper was identified as a potential climate refugium for the ten *Meconopsis* species, almost no least‐cost paths connect to this area.

## 4 DISCUSSION

For the ten *Meconopsis* species we investigated, our results show that areas of low vulnerability under future climate change are located in the central Qinghai‐Tibet Plateau (QTP proper), the eastern Himalayas, the Hengduan Mountains (HDM), and the West Qinling Mountain (WQL). A recent study by He et al. (2019) used ecological niche models to project future suitable areas for seven of the ten *Meconopsis* species we studied. They showed for five of these species areas of climatically suitability covered the HDM and WQL under future climate change, while the eastern Himalayas is climatically suitable for four *Meconopsis* species. However, unlike our results, He et al. (2019) did not show that the central QTP proper as a suitable area in the future, possibly due to differences in the research methods.

Our findings for the ten *Meconopsis* species show climate refugia under anthropogenic climate change are likely to be found in areas of low vulnerability (Figure 2, Figure S3a,b). Interestingly, these areas of predicted future climate refugia coincide with past refugia in the Tibeto‐Himalayan region (THR). Previous studies suggested past climate refugia for plant species in the Himalayas (Jia et al., 2011; Opgenoorth et al., 2010; Ren et al., 2017; Wang et al., 2010). For example, Ren et al. (2017) identified for the alpine plant *Primula tibetica* (Primulaceae) climate refugia in eastern, central and southwestern Himalayas during the LGM using both genomic analyses and ecological niche models. The HDM region is also recognized as a potential refugium for the alpine herbaceous plant *Metagentiana striata* (Gentianaceae) and the tree species *Juniperus przewalskii* (Cupressaceae) during Quaternary glaciations (Chen et al., 2008; Zhang et al., 2005). Wang et al. (2009) also asserted that the cold‐tolerant species *Aconitum gymnandrum* (Ranunculaceae) may have survived centrally on the QTP proper during the Quaternary. Moreover, our study suggests large overlaps of climate in refugia found in the central QTP proper, the eastern Himalayas, the HDM, and the WQL among time periods, that is, LGM, current and future (Figure S5). This indicates these areas may serve as long‐term climatically stable refugia and could be refugia for the ten *Meconopsis* species under anthropogenic climate change. Moreover, the ruggedness of the HDM, together with their north‐south orientation, provided refugia and short dispersal distances to favorable habitats under past climate change (Muellner‐Riehl, 2019). The presence of steep ecological gradients in the region likely also buffered against the effects of regional climate variability by facilitating range shifts (Lancaster & Kay, 2013; Muellner‐Riehl, 2019). In addition, the WQL has a complex topography where species are also likely to find environments to buffer against the effects of climate change (Xu et al., 2019). The complex topography in past refugia of the THR, which buffered against the effects of past climate change, is likely to also play a similar role in facilitating species survival during future climate change. Therefore, the regions of the central QTP proper, the eastern Himalayas, the HDM, and the WQL could potentially be future climate refugia for *Meconopsis* species under climate change.

Identifying and protecting refugia, where extinction risks are lower, is a priority for conservation under anthropogenic climate change (Hampe & Petit, 2005). However, poor habitat connectivity can prevent species from dispersing to climate refugia that could ensure their survival in the long‐term. Our results show that there are almost no dispersal routes for the ten *Meconopsis* species to the central QTP proper. We found, however, a number of dispersal routes for *Meconopsis* species to the eastern Himalayas, the HDM, and the WQL. Due to the current distributions for the ten *Meconopsis* species and poor habitat connectivity from land use and land cover modifications, potential dispersal routes to climate refugia will vary under anthropogenic climate change, consistent with the findings of Lenoir and Svenning (2015). For the currently widespread species, *M. horridula, M. integrifolia, M. lancifolia*, and *M. racemose*, potential dispersal routes are likely the HDM, the eastern Himalayas, and the WQL. The species currently found only in the eastern part of the THR, *M. punicea* and *M. quintuplinervia,* will potentially disperse to the WQL and the HDM. For those species mainly distributed on the southern edge of the THR, *M. betonicifolia*, *M. paniculata, and M. simplicifolia*, potential dispersal routes are the HDM and the eastern Himalayas. Therefore, *Meconopsis* species will likely persist in multiple climate refugia under future climate change, similar to the "microrefugia" hypothesis proposed by Wang et al. (2009) for the existence of multiple refugia in the THR for the alpine plant species *Aconitum gymnandrum* (Ranunculaceae) during the LGM.

Many alpine plant species are poor long‐distance dispersers (Herrmann et al., 2016; Morgan & Venn, 2017; Riibak et al., 2015). This is also the case for the *Meconopsis* species, as their dispersal ability by gravity‐dispersed seeds is limited to short distances (Yang et al., 2012). Hence, it is likely to be difficult for them to disperse long distances under anthropogenic climate change. Our results show that the size of the refugium on the HDM will likely be larger than others for *Meconopsis* species in the THR, and most of the potential dispersal routes to this refugium will involve relatively short‐distance dispersal. We also found that the potential HDM refugium will probably harbor more of the ten *Meconopsis* species than other identified refugia. These findings are similar to a recent study by Liang et al. (2018) that found the HDM could harbor higher species diversity under anthropogenic climate change than at present. The HDM region has complex topography and should therefore be able to buffer against the effects of future climate change for many threatened endemic species.

In this study, we used an ensemble of the six GCMs to reduce the uncertainty caused by different climate models. Furthermore, we used two RCPs (i.e., RCP 4.5 and RCP 8.5) to explore the impact of different potential future CO_2_ emission pathways on the vulnerability of the ten *Meconopsis* species. Although the exposure and vulnerability under the high CO_2_ emission pathway (i.e., RCP 8.5) are higher than those under the low CO_2_ emission pathway (i.e., RCP 4.5), regions identified as having low vulnerability under climate change are the same for the two RCPs. The ten *Meconopsis* species are mainly found in the central and eastern part of the QTP, leading to most species having a similar exposure to the impacts of climate change. Moreover, the majority of the *Meconopsis* species show similar sensitivity to bioclimatic factors, that is, a higher sensitivity to the precipitation of driest and coldest quarters than mean temperature of wettest and driest quarters. Therefore, the use of mean vulnerability for the ten *Meconopsis* species to represent the genus response to climate change seems reasonable. In saying that, as we only studied the vulnerability of ten out of the ~60 *Meconopsis* species due to low number of occurrence records for most species, we cannot rule out the possibility that increasing the number of *Meconopsis* species studied could result in predicting additional low‐vulnerability regions for the genus in the THR.

For species conservation assessments, climate‐related future threats are not often adequately accounted for (Thomas et al., 2004; Thuiller et al., 2005). The CNFA method has been proposed to assess climate change vulnerability for multiple species under anthropogenic climate change (Rinnan & Lawler, 2019). Our study found that among the ten *Meconopsis* species, *M. horridula* has relatively low vulnerability to climate change. However, the Red List of Chinese plants database (http://www.chinaplantredlist.org/) currently recognizes *M. horridula* as a NT species, while the other *Meconopsis* species are considered to be LC. Hence, current threats from land use change and over‐collection do not necessarily coincide with the threat posed by climate change, illustrating the need to include the threat from future climate change into Red List assessments.

In conclusion, the potential climate refugia for the ten *Meconopsis* species on the central QTP proper, the eastern Himalayas, the HDM, and the WQL are in regions similar to past refugia reported for other alpine plant species (Ashcroft, 2010; Ashcroft et al., 2012; Gavin et al., 2014; Tang et al., 2018). These refugia could have provided climatically suitable habitat for *Meconopsis* species to persist through past climate change and were identified as being suitable under near‐future climate change. When considering potential dispersal routes, the eastern Himalayas, the HDM, and the WQL are most likely to become future climate refugia for *Meconopsis* species, with currently widely distributed species probably being able to persist in multiple refugia. Among the refugia, the HDM is likely to be the largest climate refugia for the ten *Meconopsis* species, with species diversity increasing under climate change. Our findings are important for understanding suitable areas for alpine plants in the future and planning for conservation in mountain ecosystems under anthropogenic climate change.

## CONFLICT OF INTEREST

We declare no conflicts of interest.

## AUTHOR CONTRIBUTION

**Wen‐Ting Wang:** Conceptualization (equal); Data curation (lead); Formal analysis (lead); Funding acquisition (equal); Writing‐original draft (lead); Writing‐review & editing (lead). **Wenyong Guo:** Conceptualization (equal); Formal analysis (supporting); Writing‐review & editing (equal). **Scott Jarvie:** Conceptualization (supporting); Writing‐review & editing (equal). **Jens Christian Svenning:** Conceptualization (equal); Formal analysis (supporting); Funding acquisition (equal); Writing‐review & editing (equal).

## Supporting information

Appendix S1

## Data Availability

The cleaned occurrence records for the ten *Meconopsis* species investigated in this study: Dryad https://doi.org/10.5061/dryad.nvx0k6dpx
