# Supplementary material for: The fate of Meconopsis species in the Tibeto‐Himalayan region under future climate change
Source: Ecol Evol. 2020 Dec 28;11(2):887–99. doi: 10.1002/ece3.7096 (PMC7820157; doi:10.1002/ece3.7096)
Supplement: Supplementary file 1 — Appendix S1 [file ECE3-11-887-s001.docx]

**Supplementary materials**

**The fate of *Meconopsis* species in the Tibeto-Himalayan region under future climate change**

*Authors:*

Wen-Ting Wang ^1,2,3,4*^, Wen-Yong Guo^3,4^, Scott Jarvie^3,4^, Jens-Christian Svenning^3,4^

*Affiliations:*

^1^School of Mathematics and Computer Science, Northwest Minzu University, Lanzhou 730030, China

^2^Key Laboratory of China's Ethnic Languages and Information Technology of Ministry of Education, Northwest Minzu University, Lanzhou, Gansu 730030, China

^3^Center for Biodiversity Dynamics in a Changing World (BIOCHANGE), Department of Biology, Aarhus University, 8000 Aarhus C, Denmark

^4^Section for Ecoinformatics & Biodiversity, Department of Biology, Aarhus University, 8000 Aarhus C, Denmark

**Table S1** Number of cleaned occurrence records and assigned threat category from the Red List of Chinese Plants for ten *Meconopsis* species found in the Tibeto-Himalayan region.

| Species | Occurrence records | Red List Category |
| --- | --- | --- |
| *M. betonicifolia* | 20 | Least Concern |
| *M. horridula* | 224 | Near Threatened |
| *M.* *impedita* | 17 | Least Concern |
| *M. integrifolia* | 228 | Least Concern |
| *M. lancifolia* | 34 | Least Concern |
| *M. paniculata* | 33 | Least Concern |
| *M. punicea* | 97 | Least Concern |
| *M. quintuplinervia* | 71 | Least Concern |
| *M. racemose* | 107 | Least Concern |
| *M. simplicifolia* | 25 | Least Concern |

| **Table S2** Correlation matrix of the 19 bioclimatic variables from the WorldClim v1.4 database (<http://www.worldclim.org/>) screened for the climate vulnerability assessments of ten *Meconopsis* species in the Tibeto-Himalayan region. Values in bold are those with pairwise Pearson’s correlation coefficients above 0.80. From the correlation matrix, we remove the Pearson’s correlation between the same bioclimatic variables, e.g., bio01 and bio01. | | | | | | | | | | | | | | | | | | | |
| --- | --- | --- | --- | --- | --- | --- | --- | --- | --- | --- | --- | --- | --- | --- | --- | --- | --- | --- | --- |
|  | bio01 | bio02 | bio03 | bio04 | bio05 | bio06 | bio07 | bio08 | bio09 | bio10 | bio11 | bio12 | bio13 | bio14 | bio15 | bio16 | bio17 | bio18 | bio19 |
| bio01 |  | -0.18 | 0.23 | -0.43 | **0.94** | **0.97** | -0.36 | **0.91** | **0.92** | **0.97** | **0.98** | 0.46 | 0.52 | 0.21 | 0.20 | 0.50 | 0.27 | 0.32 | 0.14 |
| bio02 | -0.18 |  | 0.38 | 0.35 | -0.01 | -0.35 | 0.64 | -0.07 | -0.18 | -0.11 | -0.24 | -0.64 | -0.50 | -0.72 | 0.54 | -0.52 | -0.72 | -0.54 | -0.47 |
| bio03 | 0.23 | 0.38 |  | -0.70 | 0.04 | 0.28 | -0.45 | 0.19 | 0.24 | 0.07 | 0.36 | 0.24 | 0.35 | -0.28 | 0.52 | 0.33 | -0.17 | 0.33 | -0.23 |
| bio04 | -0.43 | 0.35 | -0.70 |  | -0.13 | -0.60 | **0.93** | -0.28 | -0.46 | -0.22 | -0.61 | -0.69 | -0.72 | -0.25 | -0.21 | -0.72 | -0.37 | -0.65 | -0.15 |
| bio05 | **0.94** | -0.01 | 0.04 | -0.13 |  | **0.85** | -0.05 | **0.89** | **0.86** | **0.99** | **0.86** | 0.22 | 0.29 | 0.08 | 0.21 | 0.27 | 0.12 | 0.07 | 0.09 |
| bio06 | **0.97** | -0.35 | 0.28 | -0.60 | **0.85** |  | -0.57 | **0.85** | **0.91** | **0.90** | **0.99** | 0.60 | 0.64 | 0.33 | 0.14 | 0.62 | 0.40 | 0.45 | 0.22 |
| bio07 | -0.36 | 0.64 | -0.45 | **0.93** | -0.05 | -0.57 |  | -0.22 | -0.37 | -0.16 | -0.53 | -0.78 | -0.74 | -0.48 | 0.06 | -0.75 | -0.57 | -0.75 | -0.27 |
| bio08 | **0.91** | -0.07 | 0.19 | -0.28 | **0.89** | **0.85** | -0.22 |  | 0.71 | **0.91** | **0.87** | 0.34 | 0.40 | 0.12 | 0.26 | 0.39 | 0.16 | 0.28 | -0.13 |
| bio09 | **0.92** | -0.18 | 0.24 | -0.46 | **0.86** | **0.91** | -0.37 | 0.71 |  | **0.88** | **0.91** | 0.43 | 0.50 | 0.19 | 0.16 | 0.48 | 0.28 | 0.23 | 0.36 |
| bio10 | **0.97** | -0.11 | 0.07 | -0.22 | **0.99** | **0.90** | -0.16 | **0.91** | **0.88** |  | **0.91** | 0.32 | 0.38 | 0.17 | 0.16 | 0.36 | 0.20 | 0.18 | 0.12 |
| bio11 | **0.98** | -0.24 | 0.36 | -0.61 | **0.86** | **0.99** | -0.53 | **0.87** | **0.91** | **0.91** |  | 0.56 | 0.62 | 0.24 | 0.22 | 0.60 | 0.32 | 0.43 | 0.16 |
| bio12 | 0.46 | -0.64 | 0.24 | -0.69 | 0.22 | 0.60 | -0.78 | 0.34 | 0.43 | 0.32 | 0.56 |  | **0.96** | 0.53 | -0.13 | 0.97 | 0.62 | **0.93** | 0.36 |
| bio13 | 0.52 | -0.50 | 0.35 | -0.72 | 0.29 | 0.64 | -0.74 | 0.40 | 0.50 | 0.38 | 0.62 | **0.96** |  | 0.36 | 0.09 | 1.00 | 0.49 | **0.88** | 0.27 |
| bio14 | 0.21 | -0.72 | -0.28 | -0.25 | 0.08 | 0.33 | -0.48 | 0.12 | 0.19 | 0.17 | 0.24 | 0.53 | 0.36 |  | -0.53 | 0.38 | 0.94 | 0.43 | 0.59 |
| bio15 | 0.20 | 0.54 | 0.52 | -0.21 | 0.21 | 0.14 | 0.06 | 0.26 | 0.16 | 0.16 | 0.22 | -0.13 | 0.09 | -0.53 |  | 0.05 | -0.49 | -0.12 | -0.44 |
| bio16 | 0.50 | -0.52 | 0.33 | -0.72 | 0.27 | 0.62 | -0.75 | 0.39 | 0.48 | 0.36 | 0.60 | **0.97** | 1.00 | 0.38 | 0.05 |  | 0.50 | **0.90** | 0.27 |
| bio17 | 0.27 | -0.72 | -0.17 | -0.37 | 0.12 | 0.40 | -0.57 | 0.16 | 0.28 | 0.20 | 0.32 | 0.62 | 0.49 | 0.94 | -0.49 | 0.50 |  | 0.50 | 0.68 |
| bio18 | 0.32 | -0.54 | 0.33 | -0.65 | 0.07 | 0.45 | -0.75 | 0.28 | 0.23 | 0.18 | 0.43 | **0.93** | **0.88** | 0.43 | -0.12 | 0.90 | 0.50 |  | 0.14 |
| bio19 | 0.14 | -0.47 | -0.23 | -0.15 | 0.09 | 0.22 | -0.27 | -0.13 | 0.36 | 0.12 | 0.16 | 0.36 | 0.27 | 0.59 | -0.44 | 0.27 | 0.68 | 0.14 |  |

**Table S3** The ratio of occurrence records on each land cover class to the total number of occurrence records for ten *Meconopsis* species in the Tibeto-Himalayan region.

| Land cover class | Ratio |
| --- | --- |
| Post-flooding or irrigated croplands | 0.028028 |
| Rainfed croplands | 0.2292292 |
| Mosaic cropland (50-70%) / vegetation (grassland, shrubland, forest) (20-50%) | 0.1051051 |
| Mosaic vegetation (grassland, shrubland, forest) (50-70%) / cropland (20-50%) | 0.1401401 |
| Closed to open (>15%) broadleaved evergreen and/or semi-deciduous forest (>5m) | 0.014014 |
| Closed (>40%) broadleaved deciduous forest (>5m) | 0 |
| Open (15-40%) broadleaved deciduous forest (>5m) | 0 |
| Closed (>40%) needleleaved evergreen forest (>5m) | 0.1551552 |
| Open (15-40%) needleleaved deciduous or evergreen forest (>5m) | 0 |
| Closed to open (>15%) mixed broadleaved and needleleaved forest (>5m) | 0.031031 |
| Mosaic forest-shrubland (50-70%) / grassland (20-50%) | 0.021021 |
| Mosaic grassland (50-70%) / forest-shrubland (20-50%) | 0.014014 |
| Closed to open (>15%) shrubland (<5m) | 0.014014 |
| Closed to open (>15%) grassland | 0.1591592 |
| Sparse (>15%) vegetation (woody vegetation, shrubs, grassland) | 0 |
| Closed (>40%) broadleaved forest regularly flooded - Fresh water | 0 |
| Closed (>40%) broadleaved semi-deciduous and/or evergreen forest regularly flooded - saline water | 0 |
| Closed to open (>15%) vegetation (grassland, shrubland, woody vegetation) on regularly flooded or waterlogged soil - fresh, brackish or saline water | 0 |
| Artificial surfaces and associated areas (urban areas >50%) GLOBCOVER 2009 | 0.024024 |
| Bare areas | 0.045045 |
| Water bodies | 0 |
| Permanent snow and ice | 0.02002 |

**Table S4** Pearson’s correlation coefficient of spatial vulnerability for ten *Meconopsis* species as predicted by climate-niche factor analysis based on both species range and occurrence records under future climate for the year 2070. The future climate scenarios were estimated from an ensemble of six global climate models projections under the representative concentration pathways (RCPs) 4.5 and 8.5

| Species | Pearson’s correlation | |
| --- | --- | --- |
|  | RCP 4.5 | RCP 8.5 |
| *M. betonicifolia* | 0.978 | 0.974 |
| *M. horridula* | 0.972 | 0.973 |
| *M.* *impedita* | 0.971 | 0.97 |
| *M. integrifolia* | 0.997 | 0.996 |
| *M. lancifolia* | 0.99 | 0.99 |
| *M. paniculata* | 0.959 | 0.949 |
| *M. punicea* | 0.995 | 0.994 |
| *M. quintuplinervia* | 0.996 | 0.997 |
| *M. racemose* | 0.983 | 0.981 |
| *M. simplicifolia* | 0.943 | 9.947 |


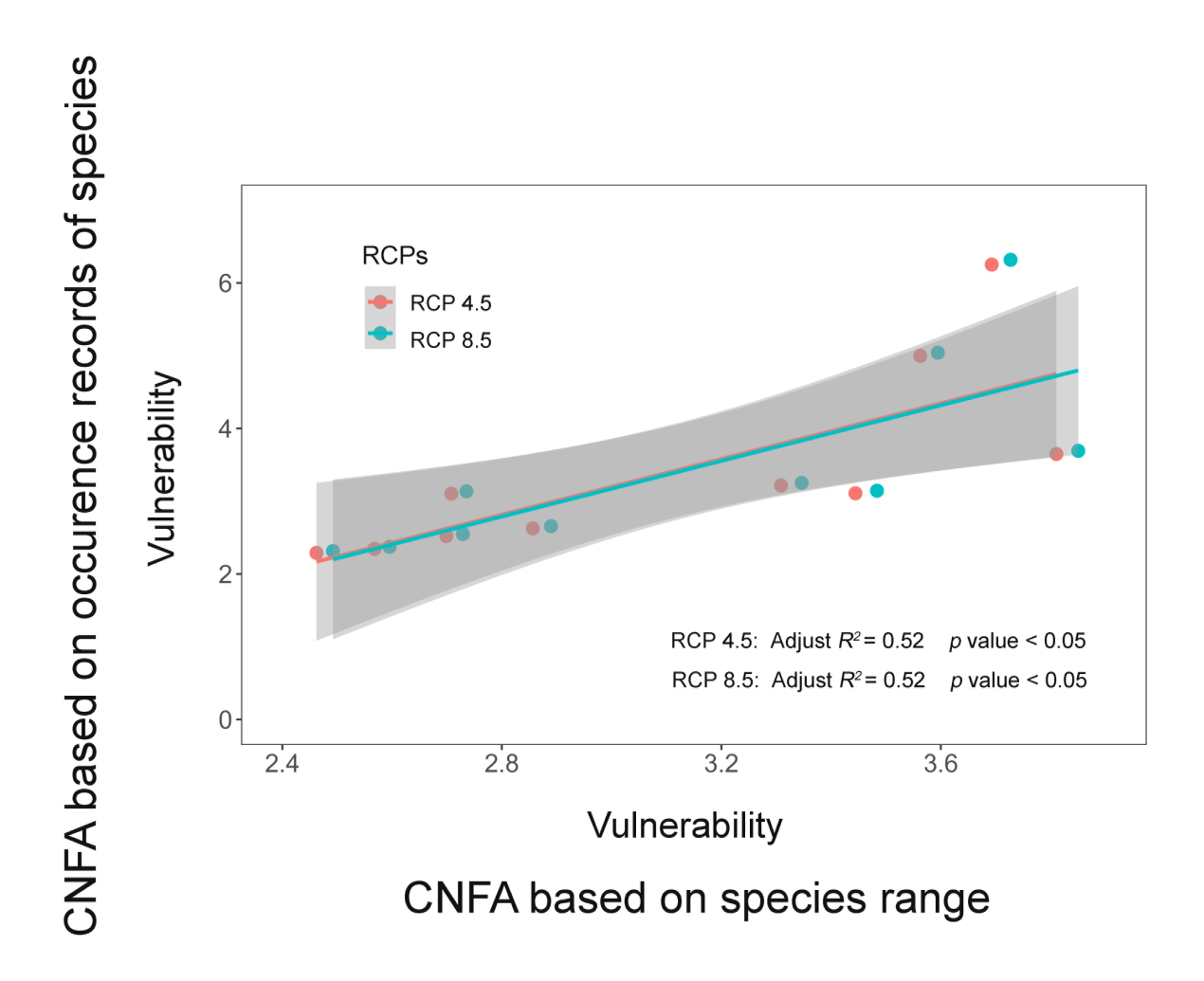


**Figure S1** Relationship of the vulnerabilities obtained through climate-niche factor analysis (CNFA) based on both species range and occurrence records for ten *Meconopsis* species for an ensemble of six global climate models projections under the representative concentration pathways (RCPs) 4.5 and 8.5 for the year 2070. The straight lines are fitted linear regressions, and the gray shadows represent the standard error.


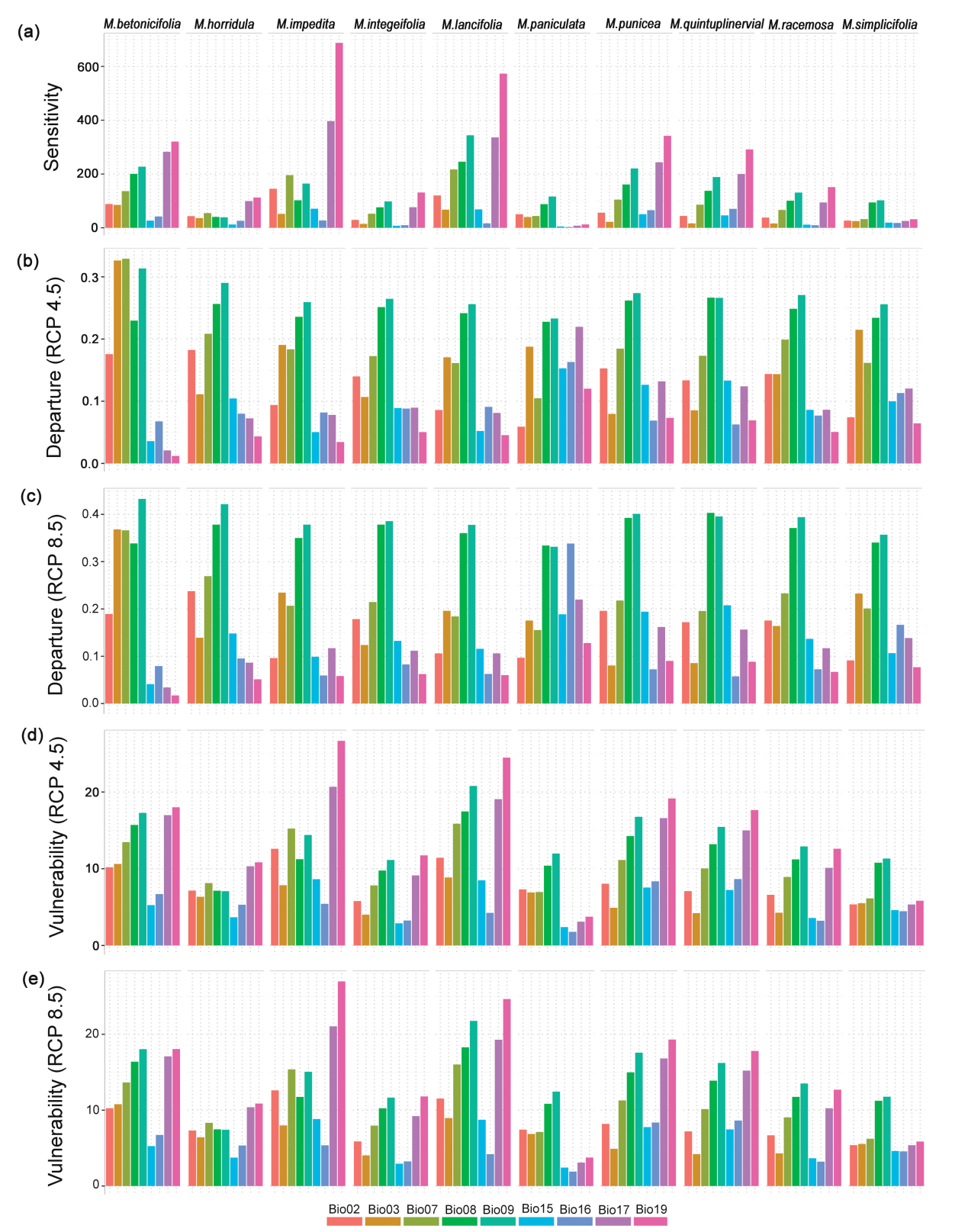


**Figure S2** The sensitivity factor (a), departure factor (b, c) and vulnerability factor (d, e) of ten *Meconopsis* species for nine bioclimatic variables calculated under future climate for the year 2070 as estimated from an ensemble of six global climate models projections under the representative concentration pathways (RCPs) 4.5 and 8.5. See Table 1 for the full names of the bioclimatic variable abbreviations.


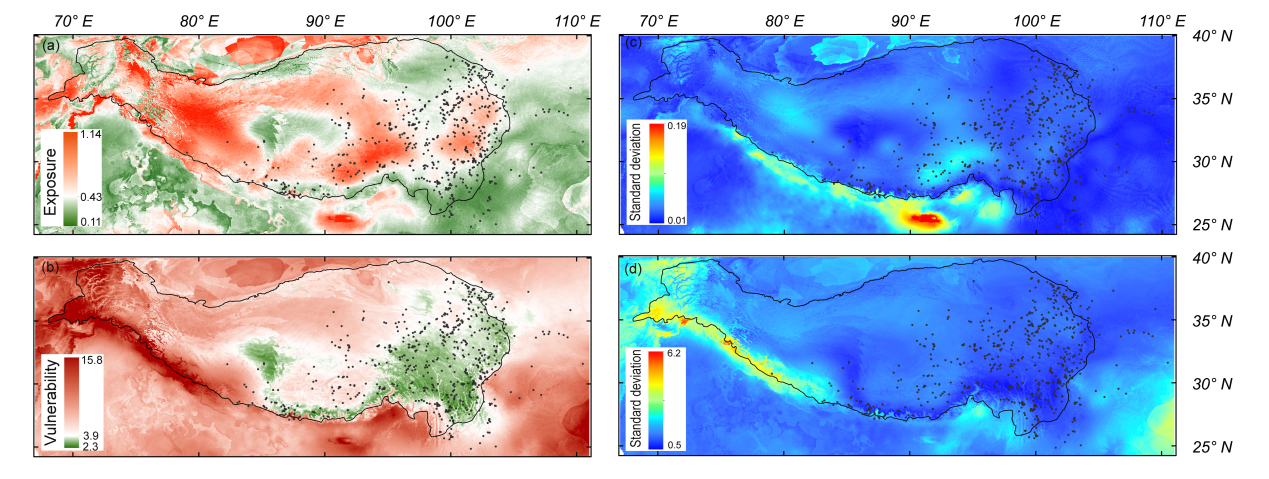


**Figure S3** Mean of predicted exposure (a), vulnerability (b) and standard deviation of predicted exposure (c), vulnerability (d) of ten *Meconopsis* species found in the Tibeto-Himalayan region and adjacent areas under future climate for the year 2070. The future climate scenario was estimated from an ensemble of six global climate models projections under the representative concentration pathway (RCP) 8.5. The black line indicates the Tibeto-Himalayan region. The black dots represent occurrence records of the ten *Meconopsis* species. For RCP 4.5, see Figure 2.


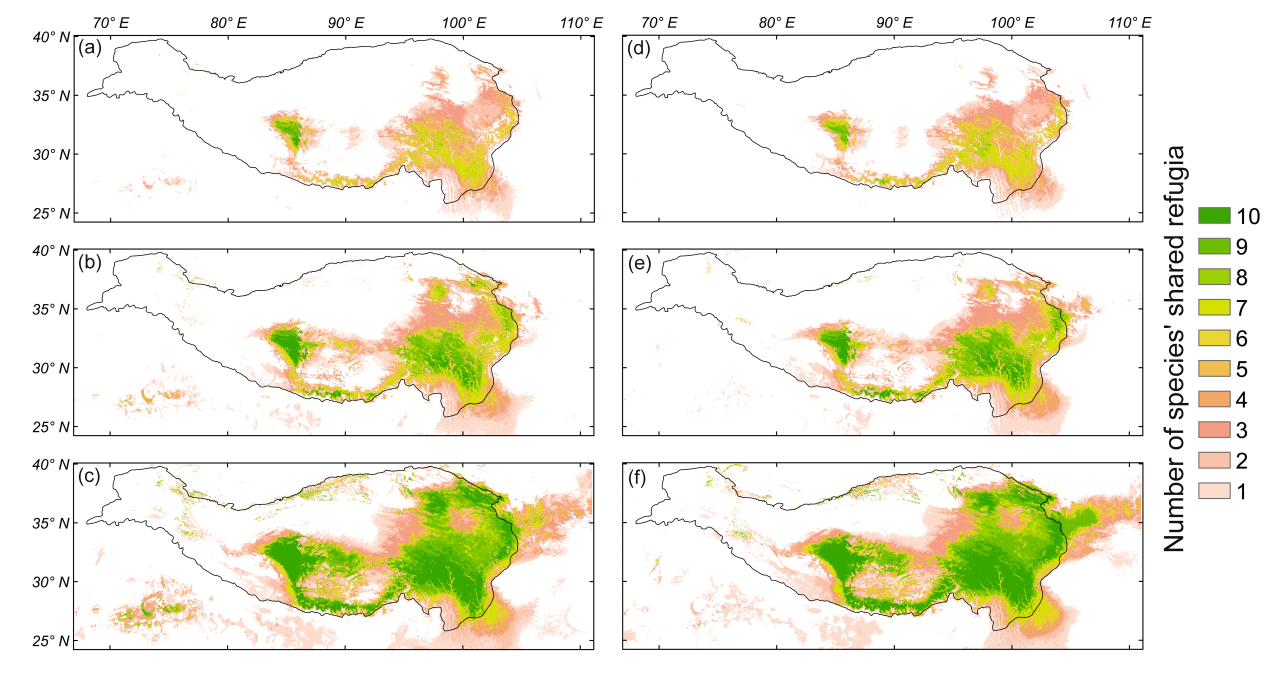


**Figure S4** Climate refugia of ten *Meconopsis* species under future climate for the year 2070 as estimated from an ensemble of six global climate models projections under the representative concentration pathways (RCPs) 4.5 and 8.5: (a) thresholds equal to 1/20 quantiles, (b) 1/10 quantiles, (c) 1/5 quantiles of spatial vulnerability value under RCP 4.5; (d) thresholds equal to 1/20 quantiles, (e) 1/10 quantiles, (f) 1/5 quantiles of spatial vulnerability value under RCP 8.5. The black line indicates the Tibeto-Himalayan region.

**
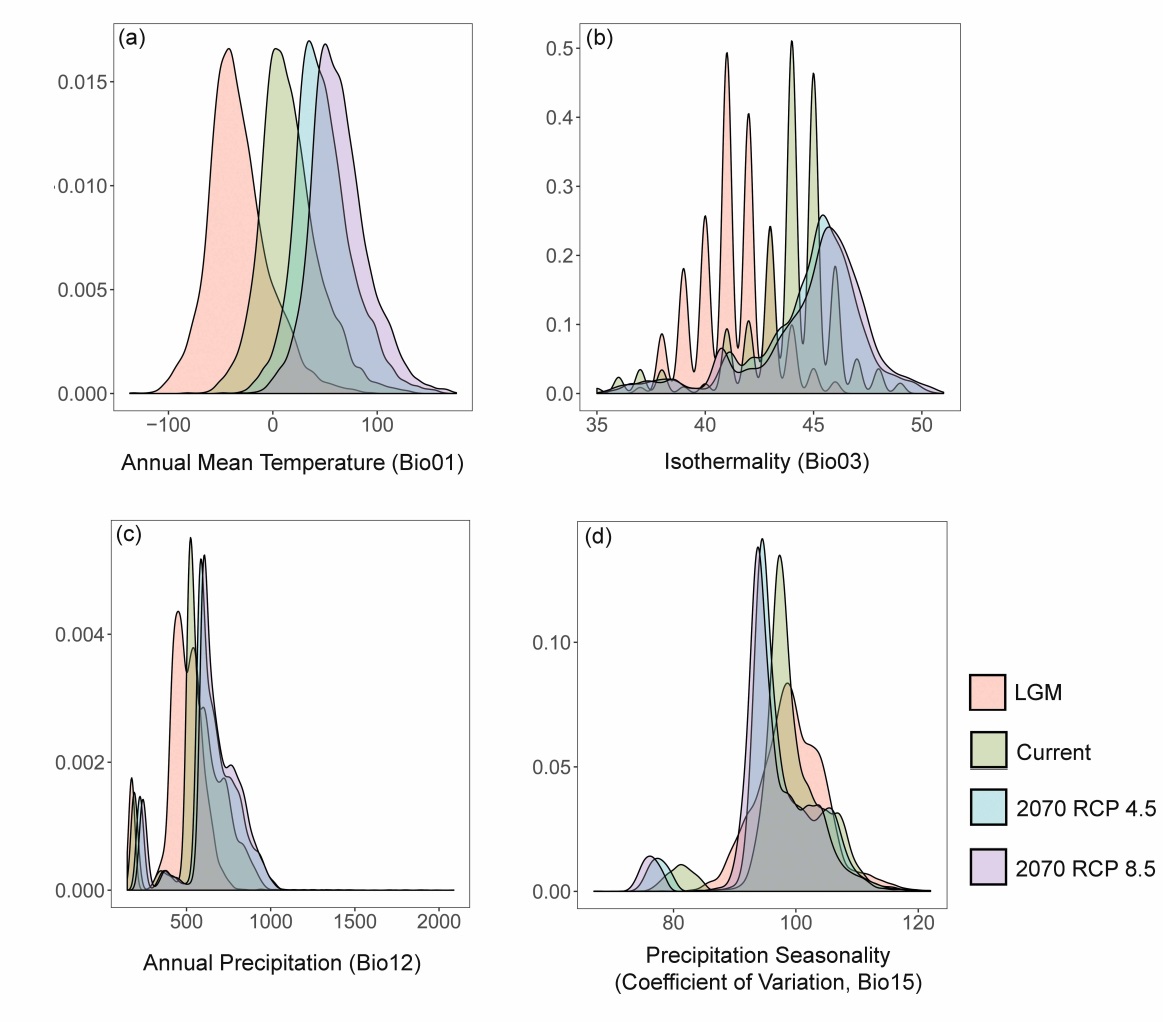
**

**Figure S5** The density overlap for annual mean temperature (a), isothermality (b), annual precipitation (c) and precipitation seasonality (d), calculated in potential climate refugia of ten *Meconopsis* species in three time frames: Last Glacial Maximum (LGM)-CCSM4, current, and a future climate for 2070. The future climate scenarios were estimated from an ensemble of six global climate models projections under two representative concentration pathways (RCPs) for 4.5 and 8.5. Bioclimatic variables came from the WorldClim database v1.4 (<http://www.worldclim.org/>) at a 2.5 arc-min spatial resolution.
